# Henoch-Schönlein Purpura Triggered by Amebiasis in a Young Female Child: A Case Report and Literature Review

**DOI:** 10.7759/cureus.90045

**Published:** 2025-08-14

**Authors:** Humam Rajha, Zaid Sawaftah, Abdelrahman Rajha, Abed Alawna, Jana Dibas, Abdulqadir J Nashwan

**Affiliations:** 1 College of Medicine, QU Health, Qatar University, Doha, QAT; 2 Department of Medicine, An-Najah National University, Nablus, PSE; 3 Faculty of Medicine, European University, Tbilisi, GEO; 4 Department of Nursing and Midwifery Research, Hamad Medical Corporation, Doha, QAT

**Keywords:** amebiasis, diarrhea, immunoglobulin a, palpable purpura, vasculitis

## Abstract

Immunoglobulin A (IgA) vasculitis, formerly known as Henoch-Schönlein purpura (HSP), is the most common form of systemic vasculitis in children, characterized by the deposition of IgA within small blood vessels. While HSP typically manifests with purpuric rash, arthritis, and gastrointestinal involvement, reports of HSP triggered by parasitic infections, such as amebiasis, are rare. This case examines the association between HSP and *Entamoeba histolytica* infection, highlighting the importance of recognizing parasitic triggers in systemic vasculitis. A 12-year-old female with no significant past medical history presented with fatigue, abdominal pain, diarrhea, hematochezia, and vomiting. She subsequently developed a non-blanching purpuric rash on her lower extremities, accompanied by joint pain and swelling. Laboratory findings revealed mild anemia, elevated inflammatory markers, and a positive stool culture for *Entamoeba histolytica*. The patient was diagnosed with HSP in association with amebiasis. Treatment involved intravenous fluids, oral prednisolone, and metronidazole. This case highlights a rare association between HSP and amebiasis, suggesting that parasitic infections should be considered potential triggers in vasculitis. A thorough diagnostic approach and timely management, including corticosteroids and antimicrobial therapy, contributed to a favorable outcome in this patient.

## Introduction

Henoch-Schönlein purpura (HSP) is the most prevalent form of systemic vasculitis in children, characterized by the deposition of immunoglobulin A (IgA)-containing immune complexes within the walls of small blood vessels. The incidence of HSP is approximately 135 per million children annually, with a significantly lower rate in adults, roughly one-tenth that of children [1]. It predominantly affects children aged between four and seven years, with about 90% of cases occurring under the age of 10, and half in those younger than six. Epidemiologically, children are most affected, with a male-to-female ratio of approximately 2:1 [2]. 

Clinically, HSP manifests across multiple organ systems, typically presenting with a symmetrical, non-tender purpuric rash, arthritis or arthralgia, and, in more severe cases, renal involvement characterized by hematuria, proteinuria, and nephrotic syndrome. Gastrointestinal involvement is also common and can include symptoms such as abdominal pain, diarrhea, and gastrointestinal bleeding [3].

Pathophysiologically, HSP involves IgA- and complement C3-containing immune complex deposition in small vessel walls, often following an upper respiratory or gastrointestinal infection, leading to leukocytoclastic vasculitis with fibrinoid necrosis and perivascular neutrophilic infiltration [4]. It is most commonly triggered by viral and bacterial infections, with upper respiratory pathogens such as *Streptococcus pyogenes*, *Staphylococcus aureus*, parainfluenza virus, and adenovirus frequently implicated [5-6]. However, reports associating HSP with parasitic infections, such as *Entamoeba histolytica*, are exceedingly rare [7].

*Entamoeba histolytica* is a protozoan parasite transmitted via the fecal-oral route, prevalent in areas with inadequate sanitation. While most infections are asymptomatic, they can cause amebic colitis, dysentery, and, in severe cases, extraintestinal manifestations such as liver abscesses. Globally, it accounts for approximately 50 million symptomatic cases and up to 100,000 deaths annually [8]. Previous case reports have described HSP triggered by parasitic infections, including giardiasis and toxocariasis, with amebiasis being an especially rare trigger [9].

Herein, we present the case of a 12-year-old female who presented with fatigue, abdominal pain, diarrhea, hematochezia, and persistent vomiting. She subsequently developed a non-blanching purpuric rash localized to the lower extremities, accompanied by joint swelling and pain. Laboratory findings revealed mild anemia, leukocytosis, and a stool culture positive for *Entamoeba histolytica*. The patient was diagnosed with HSP in association with amebiasis, highlighting a rare yet important consideration in the differential diagnosis of vasculitis in children.

## Case presentation

Case history and examination

**Figure 1:**
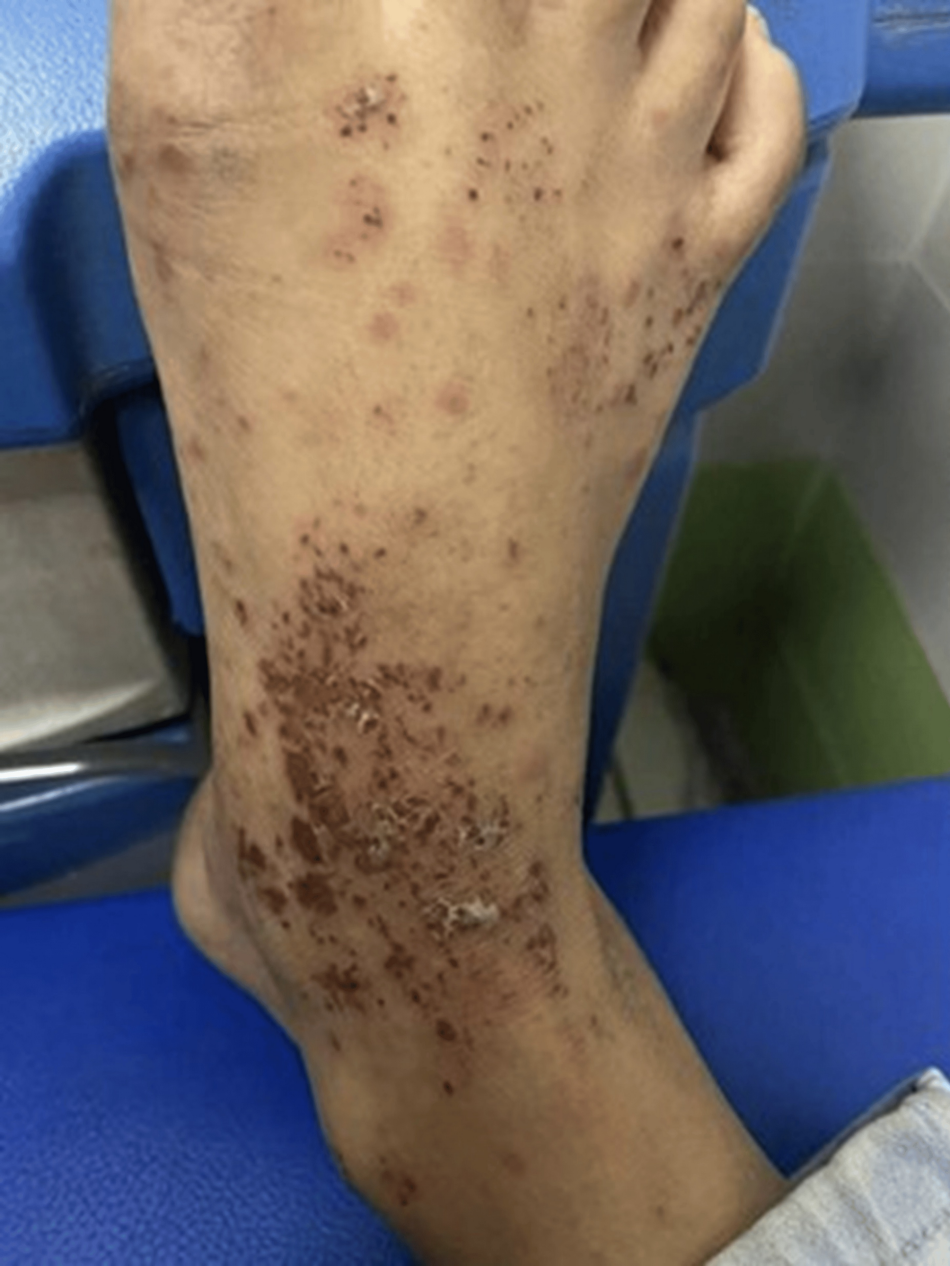
Palpable purpura and maculopapular lesions on the foot of a pediatric patient with IgA vasculitis. Clinical photograph showing the dorsal and medial aspects of the foot with multiple scattered erythematous maculopapular lesions, some progressing to purpura. These findings are characteristic of small vessel vasculitis. Notably, areas of excoriation and crusting are visible, likely secondary to pruritus and scratching.

A 12-year-old female with no significant past medical history presented with a constellation of symptoms, including abdominal pain, diarrhea, hematochezia, and persistent vomiting of all ingested food. Three days after the onset of gastrointestinal symptoms, she also developed a localized to her lower extremities, associated with swelling and joint pain. Upon physical examination, the patient appeared to be in pain and exhibited signs of dehydration, including dry mouth, sunken eyes, and reduced skin turgor. She had a blood pressure of 90/60 mmHg, a heart rate of 88 bpm, a respiratory rate of 18 breaths/minute, and an oxygen saturation of 98% on room air. Abdominal examination revealed diffuse tenderness without guarding or rebound. Musculoskeletal examination showed swelling, warmth, and tenderness in both knees and ankles. Despite her discomfort, she remained alert, oriented, and afebrile, with stable oxygen saturation. Her blood pressure was reduced, but her heart rate remained within normal limits. Further examination revealed swelling and pain in both lower and upper limbs, and a petechial rash on her buttocks, legs, and feet, which had progressively worsened, leading to the development of palpable purpura (Figure 1).

Methods (differential diagnosis, investigations, and management)

Given the patient's clinical presentation, differential diagnosis included HSP, post-infectious vasculitis, meningococcemia, and bacterial or parasitic gastroenteritis. Further investigations were initiated, including laboratory tests such as a complete blood count (CBC), inflammatory markers, coagulation profile, kidney function tests, serum IgA, stool culture, and stool examination for occult blood. The results revealed mild anemia, elevated inflammatory markers such as C-reactive protein (CRP) and erythrocyte sedimentation rate (ESR), which are typically high in HSP due to systemic vasculitis and cytokine-mediated endothelial injury, which stimulate the acute-phase response. In addition, leukocytosis and the presence of* Entamoeba histolytica*, as identified on stool culture, along with a positive fecal occult blood test. Serum IgA was mildly elevated, supporting the diagnosis, while kidney function remained within normal limits, consistent with the absence of renal involvement (Table 1). Serial abdominal ultrasound examinations showed no significant findings. Based on the clinical presentation and the EULAR/PRINTO/PRES 2010 classification criteria [6], the patient was diagnosed with HSP, characterized by non-thrombocytopenic palpable purpura and abdominal pain, in the context of concurrent amebiasis. Treatment included supportive care with IV fluids, IV pantoprazole (30 mg), oral prednisolone (5 mg), and oral metronidazole (400 mg) targeting the *Entamoeba histolytica* infection.

**Table 1:** Laboratory test results and analysis. This covers the complete blood count, inflammatory markers, blood chemistry, stool analysis, and coagulation profile. eGFR, estimated glomerular filtration rate

Complete blood count	Result	Normal range
Hemoglobin (HgB)	10.1 g/dL	12.0-15.5 g/dL
Hematocrit (HCT)	31%	34%-44%
Mean cell volume (MCV)	71 fL	80-100 fL
White blood cells (WBCs)	16,000 cells/μL	4,000-11,000 cells/μL
Neutrophil	87%	40%-70%
Absolute neutrophil count (ANC)	12,900 cells/μL	1,500-8,000 cells/μL
Platelets count	700,000 cells/μL	150,000-450,000 cells/μL
Anti-Inflammatory markers		
C-reactive protein (CRP)	109 mg/L	0-10 mg/L
Erythrocyte sedimentation rate (ESR)	186 mm/hour	0-29 mm/hour
Blood chemistry		
Aspartate aminotransferase (AST)	45.2 U/L	0-37 U/L
Alanine transaminase (ALT)	29 U/L	0-33 U/L
Lactate dehydrogenase (LDH)	500 U/L	207-414 U/L
Blood urea nitrogen	6 mg/dL	6-24 mg/dL
Creatinine, serum	0.54 mg/dL	0.5-0.9 mg/dL
Stool analysis		
Fecal occult blood test	Positive	Negative
Consistency	Diarrhetic	Normal (Formed)
WBCs	15-20/hpf	0-5/hpf
Trophozoite	++	Negative
Cyst	++	Negative
Entamoeba histolytica	++	Negative
Mucus	+++	Negative
Coagulation profile test		
Activated partial thromboplastin time (APTT)	37 seconds	25-35 seconds
Prothrombin time (PT)	15.5 seconds	11-14 seconds
Fibrinogen	341 mg/dL	200-400 mg/dL
eGFR	>90 mL/minute/1.73 m²	>90 mL/minute/1.73 m²

Outcomes and follow-up

Over the following days, the patient exhibited clinical improvement, with a reduction in abdominal pain and diarrhea. Follow-up laboratory tests revealed a decrease in inflammatory markers and an overall improvement, indicating a positive response to treatment (Table 2). Ultrasound imaging showed no additional complications. The patient’s erythematous maculopapular lesions and palpable purpura also began to heal, indicating a favorable systemic response. Follow-up visits were scheduled to ensure ongoing management and to monitor for potential complications. She was also advised on lifestyle modifications, including practicing good hygiene, drinking safe water, eating well-cooked food, avoiding contaminated food, adhering to medications, and attending regular medical check-ups.

**Table 2:** Follow-up laboratory test results (post-treatment). eGFR, estimated glomerular filtration rate

Test category	Result (Day 1)	Result (Day 6)	Normal range
Complete blood count			
Hemoglobin (HgB)	10.1 g/dL	11.8 g/dL	12.0-15.5 g/dL
Hematocrit (HCT)	31%	33%	34%-44%
Mean cell volume (MCV)	71 fL	75 fL	80-100 fL
White blood cells (WBCs)	16,000 cells/μL	12,000 cells/μL	4,000-11,000 cells/μL
Neutrophils	87%	75%	40-70%
Absolute neutrophil count (ANC)	12,900 cells/μL	8,800 cells/μL	1,500-8,000 cells/μL
Platelet count	700,000 cells/μL	450,000 cells/μL	150,000-450,000 cells/μL
Anti-inflammatory markers			
C-reactive protein (CRP)	109 mg/L	45 mg/L	0-10 mg/L
Erythrocyte sedimentation rate (ESR)	186 mm/hour	120 mm/hour	0-29 mm/hour
Blood chemistry			
Aspartate aminotransferase (AST)	45.2 U/L	37 U/L	0-37 U/L
Alanine transaminase (ALT)	29 U/L	27 U/L	0-33 U/L
Lactate dehydrogenase (LDH)	500 U/L	450 U/L	207-414 U/L
Stool analysis			
Fecal occult blood test	Positive	Negative	Negative
Consistency	Diarrhetic	Normal (Formed)	Normal (Formed)
WBCs	15-20/hpf	5-10/hpf	0-5/hpf
Trophozoite	++	Negative	Negative
Cyst	++	Negative	Negative
Entamoeba histolytica	++	Negative	Negative
Mucus	+++	Negative	Negative
Coagulation profile test			
Activated partial thromboplastin time (APTT)	37 seconds	35 seconds	25-35 seconds
Prothrombin time (PT)	15.5 seconds	14.2 seconds	11-14 seconds
Fibrinogen	341 mg/dL	310 mg/dL	200-400 mg/dL
Estimated GFR	>90 mL/minute/1.73 m²	>90 mL/minute/1.73 m²	>90 mL/minute/1.73 m²
Serum IgA	4.3 g/L	4.0 g/L	0.7-4.0 g/L

## Discussion

IgA vasculitis, also known as HSP or anaphylactoid purpura, is the most common form of systemic vasculitis in children, with around 90% of cases occurring in individuals under 10 years of age [10]. The disease typically affects children between the ages of three and 15 years, with peak incidence at six years [11]. It is slightly more prevalent in males, with reported male-to-female ratios ranging from 1.2:1 to 1.8:1 [12]. Clinically, IgA vasculitis is characterized by a distinctive tetrad of symptoms: a purpuric skin rash, arthralgia, abdominal pain, and renal involvement [13].

Due to its systemic involvement, HSP can lead to significant complications, primarily affecting the kidneys and gastrointestinal system. Renal involvement, such as IgA vasculitis nephritis, may progress to nephrotic syndrome or end-stage kidney disease, sometimes requiring dialysis or transplantation [14]. Gastrointestinal issues, including intussusception, bleeding, and bowel perforation, can be exacerbated by infections like amoebiasis [15]. Less common complications include seizures, central nervous system (CNS) bleeding, pulmonary hemorrhage, and pleural effusion [15]. Details of the complications are summarized in Table 3 [16].

**Table 3:** Systemic involvement and clinical manifestations of Henoch-Schönlein purpura (HSP). Source: [18].

Involved systems	Clinical manifestations
Dermatological (~100% of cases)	Symmetrically distributed palpable purpura, typically found in the lower extremities, buttocks, and other regions that experience pressure or constriction (i.e., areas affected by clothing)
Musculoskeletal (~75% of cases)	Arthritis/Arthralgia, most commonly involving the knees and ankles
Gastrointestinal (~60% of cases)	Colicky abdominal pain, nausea and vomiting, melena or hematochezia, intussusception
Renal (~20% to 50% in children; 50% to 80% in adults)	Nephritic or nephrotic syndrome

IgA vasculitis exhibits a seasonal pattern, with a higher incidence in the fall, winter, and spring, likely due to the increased frequency of infections during these periods. Summer consistently reports the lowest case rates [17]. Despite extensive research, the precise cause of HSP remains unclear. However, it is widely hypothesized that preceding upper respiratory or gastrointestinal infections may contribute to the triggering of the disease. These infections are thought to induce an overproduction of IgA antibodies, which form immune complexes that preferentially deposit in small blood vessels, especially in post-capillary venules [18]. This leads to the activation of the complement system and the recruitment of neutrophils, initiating an inflammatory response that primarily affects the gastrointestinal, musculoskeletal, dermatological, and renal systems, resulting in the clinical features of HSP [19].

Although a multitude of bacterial and viral infections have been associated with the disease, concomitant parasitic infections with HSP are extremely rare [20]. Ergür et al. reported 35 cases linking parasites to HSP, with merely 3 identifying larvae by *Entamoeba histolytica* [20]. Through our literature review, we identified three other case reports of HSP with amoebiasis [3,21,22], suggesting that while the association is possible, it is exceptionally uncommon in the pediatric population. Reported incidence of HSP varies globally from 10 to 20 cases per 100,000 children per year, with parasitic-associated cases representing only a fraction of this [10].

Amoebiasis is an intestinal infection caused by the parasite *Entamoeba histolytica*, primarily transmitted through the ingestion of food or water contaminated with *Entamoeba histolytica* cysts [23]. While the condition is more common in men, exposure rates are similar between genders [24]. The clinical presentation varies widely, with approximately 90% of cases being asymptomatic, while 1% progress to the invasive form, which can lead to severe complications like abscesses [24,25]. Intestinal amoebiasis, the most common type, results from mucosal invasion of the cecum or colon, leading to symptoms such as abdominal pain and dysentery [26]. Extraintestinal amoebiasis, including hepatic and pulmonary involvement, can be life-threatening if left untreated [27]. Diagnosis typically relies on a combination of parasitological, immunological, and molecular techniques [23,24]. Microscopic examination of stool or tissue samples is commonly used, but distinguishing *Entamoeba histolytica* from the non-pathogenic *Entamoeba dispar* can be challenging due to their morphological similarities [23]. Immunological techniques, such as enzyme-linked immunosorbent assays (ELISA), offer improved specificity but exhibit variable sensitivity, particularly in differentiating species [24]. Additionally, gene editing-based molecular tests provide excellent sensitivity and specificity; however, cost and limited accessibility remain significant barriers in endemic areas [24]. There is a critical need for rapid, affordable diagnostic tests, especially in developing countries where amoebiasis is prevalent, as effective treatment is contingent upon accurate diagnosis [23].

From a practical standpoint, differentiating *Entamoeba histolytica* from morphologically identical *Entamoeba dispar *is essential, as the latter is non-pathogenic and does not require treatment. This distinction typically requires antigen detection assays or polymerase chain reaction (PCR), as microscopy alone cannot reliably separate the two [25]. Clinically, *Entamoeba histolytica* infection is more likely to present with invasive disease, manifesting as dysentery, abdominal pain, and, in severe cases, extraintestinal spread, whereas *Entamoeba dispar* colonization is generally asymptomatic [26]. Other protozoal or bacterial causes of dysentery, such as Giardia lamblia, Shigella, or Salmonella, often differ by stool characteristics, incubation period, and associated systemic signs, underscoring the importance of targeted diagnostic testing to confirm the causative pathogen and guide management [27].

Similar to this case report, previous case reports on HSP with amoebiasis lack information on temporal precedence, and thus the causal relationship between the two is ambiguous [23-24]. It is plausible that an infectious trigger can activate the immune system, leading to flares of IgA vasculitis. However, the impaired mucosal immunity in this autoimmune disease can also make patients susceptible to parasitic infections.

While serum IgA levels were obtained in this case and supported the diagnosis, circulating immune complex measurement and biopsy with immunofluorescence, tests that can further confirm the characteristic IgA deposition in HSP, were not performed. This represents a limitation of the present report and is acknowledged accordingly.

Regardless of the causal agent or whether the association was incidental, the management approach remains consistent. Our 12-year-old patient received treatment similar to that described by several authors. The management of HSP primarily involves supportive care; in this case, intravenous fluids were administered to address dehydration. Consistent with prior reports, corticosteroids were employed for gastrointestinal and joint symptoms and tapered as the patient's condition improved. Oral prednisolone (5 mg) was prescribed due to the inflammatory nature of HSP. Additionally, metronidazole (400 mg) was initiated after *Entamoeba histolytica* was detected in the stool culture. Corticosteroids remain a key treatment for severe gastrointestinal and joint involvement in HSP, while metronidazole is the drug of choice for amoebiasis [28,29].

## Conclusions

This case highlights the rare association between HSP and amebiasis, underscoring the importance of considering parasitic infections as potential triggers for systemic vasculitis, particularly in endemic regions. Although the exact pathophysiological link between HSP and *Entamoeba histolytica* remains unclear, this report underscores the need for a comprehensive diagnostic approach in pediatric patients presenting with atypical gastrointestinal symptoms alongside vasculitic manifestations. Prompt diagnosis and early intervention, including supportive care, corticosteroids, and targeted antimicrobial therapy, contributed to a favorable outcome in this patient. Further research is warranted to elucidate the mechanisms driving the association between parasitic infections and IgA vasculitis, as well as to optimize management strategies for these rare, coexisting conditions.
